# Policies that limit youth access and exposure to tobacco: a scientific neglect of the first stages of the policy process

**DOI:** 10.1186/s12889-019-7073-x

**Published:** 2019-06-26

**Authors:** Thomas G. Kuijpers, Anton E. Kunst, Marc C. Willemsen

**Affiliations:** 1 Department of Health Promotion, CAPHRI Care and Public Health Research Institute, Maastricht University, P. Debyeplein 1, 6229 HA Maastricht, The Netherlands; 2 Department of Public Health, Academic Medical Center, University of Amsterdam, Amsterdam, The Netherlands; 3 Trimbos Institute, Netherlands Institute of Mental Health and Addiction, Utrecht, The Netherlands

**Keywords:** Tobacco control, Public policy, Advocacy, Prevention, Youth access, Policy process, Policy making

## Abstract

**Background:**

Policymakers can adopt and implement various supply-side policies to limit youth access and exposure to tobacco, such as increasing the minimum age of sale, limiting the number or type of tobacco outlets, or banning the display of tobacco products. Many studies have assessed the impact of these policies, while less is known about the preceding policy process. The aim of our review was to assess the available evidence on the preceding process of agenda setting, policy formulation, and policy legitimation.

**Methods:**

A systematic literature search was conducted using the PubMed and the Social Sciences Citation Index databases. After selection, 200 international peer-reviewed articles were identified and analyzed. Through a process of close reading, evidence based on scientific enquiry and anecdotal evidence on agenda setting, policy formulation and policy legitimation was abstracted from each article.

**Results:**

Scientific evidence on the policy process is scarce for these policies, as most of the evidence found was anecdotal. Only one study provided evidence based on a scientific analysis of data on the agenda setting and legitimation phases of policy processes that led to the adoption of display bans in two Australian jurisdictions.

**Conclusion:**

The processes influencing the adoption of youth access and exposure policies have been grossly understudied. A better understanding of the policy process is essential to understand country variations in tobacco control policy.

**Electronic supplementary material:**

The online version of this article (10.1186/s12889-019-7073-x) contains supplementary material, which is available to authorized users.

## Background

Youth smoking continues to be a widespread public health problem. Globally, an estimated 7.0% of children aged 13–15 smoke; the Americas (13.0%) and the European region (9.8%) demonstrate the highest prevalence of smoking among children [1]. Youth smoking can be prevented in various ways. One of the strategies is to reduce youth access to tobacco products. The policy most often used to achieve this reduction of access is raising the minimum age-of-sale for the purchase of tobacco. Most countries have implemented this policy, in line with the recommendations of Article 16 of the Framework Convention on Tobacco Control (FCTC), which aims to reduce sales to and by minors [2]. Reviews examining the effectiveness of age-of-sale policies report mixed and inconclusive findings and urge the consideration of enforcement conditions and personal characteristics [3–7]. A reduction in illegal sales to minors does not necessarily mean that youth tobacco consumption is also decreased, because minors can often still access tobacco through social sources [8]. This is one of the reasons some authors have concluded that age-of-sale policies are ineffective [9]. Others conclude that such policies may be effective, as long as they are well enforced [10].

Youth access to tobacco may also be reduced by limiting the number or type of tobacco outlets. Tobacco retailing throughout the world is completely normalized, and “tobacco can be sold openly, from virtually any business” [11]. Policy measures directly limiting the number of tobacco outlets, for example, to specialized shops, have rarely been adopted. Thus far, only the Hungarian government has done so, substantially reducing the number of outlets from 42,000 to 7000, with the explicit aim to decrease smoking prevalence [12]. It can be argued from the perspective of economic theory that a higher tobacco retail density leads to increased tobacco consumption due to increased availability and reduced retrieval costs [13]. However, because policies that reduce the number of sale outlets are rarely adopted, there is limited data on their effectiveness. Some, but not all, studies on this subject have reported positive associations between tobacco outlet density and smoking behavior [14–16]. However, because the studies all used observational research designs, causality cannot be inferred [17].

Next to increasing the age of sale and limiting the absolute number of outlets, policymakers may choose to ban the sale of tobacco from certain types of retail outlets. Sale restrictions may be related to the primary function of the sale outlet, which can be in conflict with tobacco sales, such as in the case of pharmacies [18]. Another option is a ban on tobacco vending machines, which may offer easy access of tobacco to minors. To address this issue, Article 16 of the FCTC recommends putting into place a ban on vending machines, or at least some restrictions on accessibility [2]. Many countries have addressed this issue; a total of 89 countries worldwide now have a ban on tobacco vending machines [19].

Directly limiting the number of tobacco retailers may be a step too far for policymakers, which is perhaps indicated by the small number of countries that have adopted this policy measure thus far. An alternative option for policymakers may be to consider banning tobacco displays at points of sale, which will not reduce physical access but aims to reduce exposure to pro-smoking messages at points of sale. Studies and reviews have demonstrated positive associations between exposure to tobacco displays and youth smoking behavior and susceptibility [20–24]. A growing number of countries have adopted a display ban [22, 25, 26], and evaluations of the countries that have implemented this ban suggest that it helps to denormalize smoking [27–29].

While there is an emerging body of literature on the effectiveness of the various policies that limit youth access and exposure to tobacco, less is known about the preceding policy process that leads to their adoption by policymakers. In fact, most public health research is carried out without considering the policy process at all [30]. Public health advocates and professionals who want to effectively use the political arena need to have at least a basic understanding of how policymaking works [31]. The more thoroughly this process is examined, the better these individuals can anticipate constraints and opportunities for policy change [32].

There are several theories that may be used to study the preceding process of policymaking up until policy adoption, such as Kingdon’s three streams [33], the punctuated equilibrium theory [34], the advocacy coalition framework [35], theories on multilevel governance [36], theories on policy transfer [37, 38] and others. These theories focus on different aspects of the policymaking process, which are relevant to different stages of the policymaking process. Cairney (2012) breaks down the policy process into the following six stages: agenda setting, policy formulation, legitimation, implementation, evaluation, and policy maintenance, succession or termination [39]. In the current paper, we are interested in the first three stages, as these are relevant to the adoption of policies. Agenda setting refers to the identification of policy problems (e.g., a high level of youth smoking). Formulation refers to the selection of appropriate solutions for the policy problem (e.g., an age-of-sale policy). Legitimation refers to ensuring that the chosen policy has enough political and public support. While much is known about the impacts of policy, considerably less is known about its antecedents [40]. A better insight into the stages up until policy adoption is of vital importance for advocates that wish to foster tobacco control policy.

The aim of this paper is to assess the scientific evidence on the first three stages of the policymaking process of raising the legal age for the purchase of tobacco, limiting the number or type of tobacco outlets, and banning tobacco displays at points of retail. We will assess the quantity and quality of the evidence that can be found in the international scientific literature.

## Methods

We conducted a literature search to find evidence on the agenda setting, policy formulation and policy legitimation stages of the policy cycle for the three policies under study. The search strategy was informed by a quick scan of the literature and by a priori inspection of the case of the Netherlands. In this preparatory step, we examined Dutch parliamentary documents about the emergence of tobacco control legislation from 1995 onwards. In addition, we interviewed a member of parliament, a civil servant, and an academic expert, and questions were sent by e-mail to international tobacco control experts. These sources provided us with relevant insights into the policymaking process of the three policies. We translated this knowledge into keywords for our search strategy (see the Additional file 1).

We conducted a literature search using the PubMed and Social Sciences Citation Index (SSCI) databases. Whereas the first database predominantly covers biomedical journals, the SSCI covers a wide range of 176 political science journals. We searched for articles published in peer-reviewed journals up to March 2016. We set no publication date requirement for the articles to be included because countries differed in the timing of policy adoption.

### Screening

The search yielded 507 references to international peer-reviewed articles. After removing 145 duplicates, 362 articles remained for title and abstract screening. The selection of studies was performed in two stages by two reviewers: TGK and Paulien Nuyts (a project member). During the first selection stage, the titles and abstracts of the selected studies were screened by both reviewers to select appropriate studies for full text screening. During the second stage, the full texts were assessed to abstract relevant evidence about the first three stages of the policy process. Because of limited time, the full-text screening was completed by TGK after a random sample of 20 articles (10%) had been screened by both reviewers to test and fine tune the eligibility criteria, as well as to ensure consensus.

**Fig. 1:**
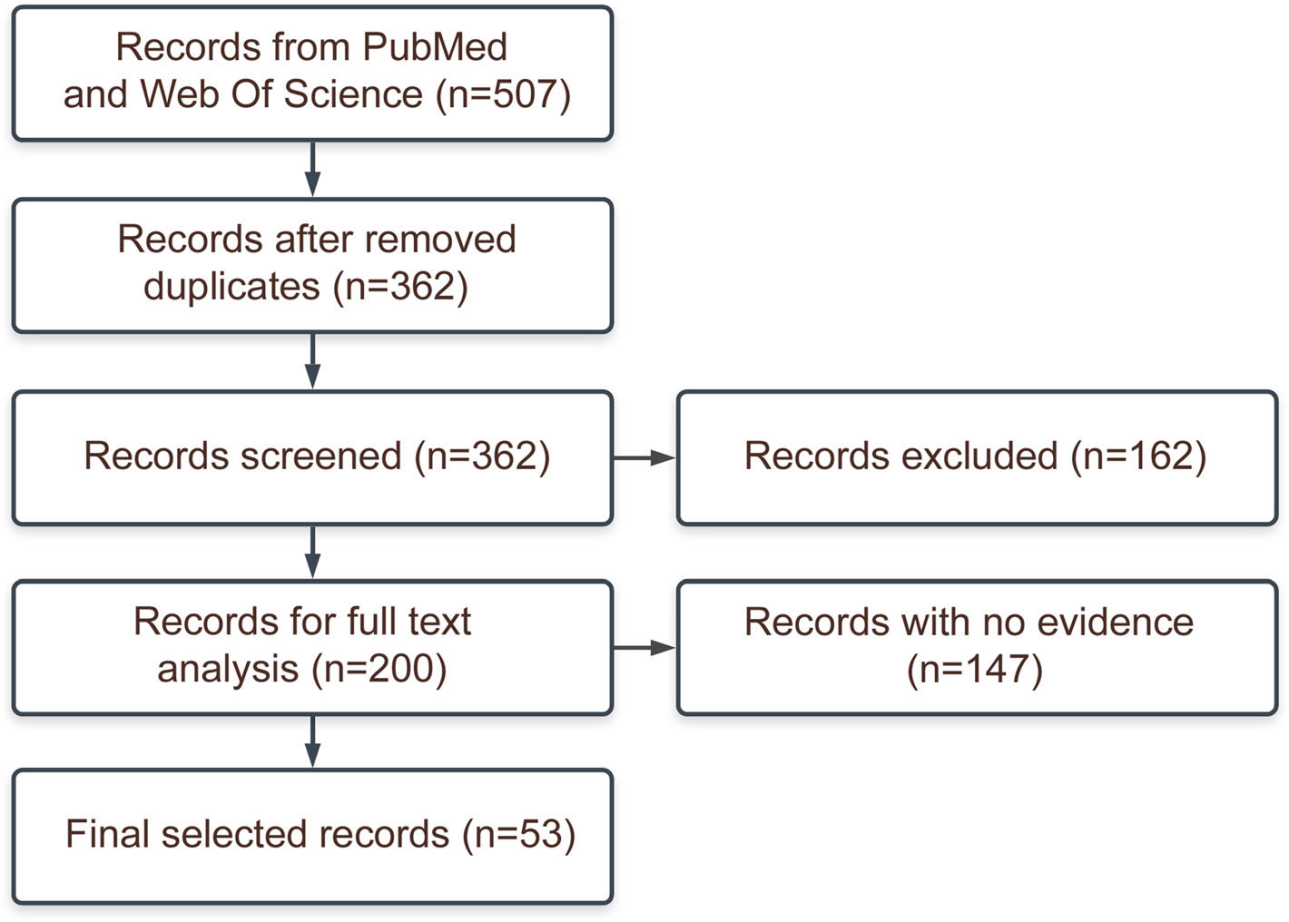
PRISM flowchart diagram of included articles

The title and abstract screening criteria were as follows: an article should 1) be written in English, 2) have a full text available and 3) concern one of the three policies of interest (i.e., raising the age of sale, limiting the number or type of sale outlets and banning tobacco displays) or broader topics such as youth access/availability. A total of 138 articles were not related to any of the three selected policies, 15 articles had no full text available, and 9 articles were not written in English. We checked whether the non-English articles mainly focused on the first three stages of the policymaking process by reading the English abstracts, and concluded this was not the case. The remaining 200 articles were analyzed to determine whether they presented evidence on any of the first three stages of the policymaking process (see data extraction below) (Fig. 1)

.

### Data extraction

Because this review is realist inspired, we followed the Realist And Meta-narrative Evidence Synthesis: Evolving Standards (RAMESES) guidelines for data extraction and appraisal [41]. Both reviewers appraised the contribution of evidence in terms of both rigor and relevance. In terms of rigor (i.e., judging the credibility and trustworthiness of evidence), a dichotomy was made between “anecdotal evidence,” such as author accounts in the introduction and discussion sections (which could be considered “thin” evidence), and evidence resulting from scientific analyses employing a scientific method (which could be considered “thick” evidence). Evidence was considered relevant if it explicitly described a causal link between a certain determinant and the adoption one of the three selected policies (e.g., the enactment of a ban on vending machines in response to the federal Synar Amendment of the United States).

Subsequently, the 200 articles were assessed by the first author alone. Evidence was deemed relevant if it met the following eligibility criteria referring to the first three stages of the policy process: 1) agenda setting: the paper provides information on the process of acknowledging an issue as a policy problem 2) policy formulation: the paper provides information on the process of formulating a policy in response to a problem, and 3) policy legitimation: the paper provides information on the process of legitimating the choice for a specific type of policy. We further extracted the title of the article, the full names of the authors, the year of publication, the main focus of the article *(“Agenda setting/policy formulation/legitimation”, “Enforcement/compliance”, “Effectiveness of policy”, “Industry misconduct”* or *“Other”)* and the policy measure the evidence was related to (*“Raising the age of Sale”, “Limiting number or type of tobacco outlets”* or *“Banning tobacco displays”*).

## Results

We found 74 pieces of evidence in 53 articles that were related to one or more of the three policy stages of interest. Fifty-two pieces of evidence were related to raising the age of sale, 15 were related to limiting the number or type of tobacco outlets, and 7 were related to banning tobacco displays. One article offered a systematic analysis of data, whereas the other 52 articles gave anecdotal author accounts. A summary of the findings for each policy can be found in Table 1. All evidence can be found in the Additional file 2.

**Table 1:** Pieces of evidence by policy and main focus of article

	Raising age of sale	Limiting number or type of tobacco outlets	Banning tobacco displays	Total
Main research focus of article				
Agenda setting/formulation/legitimation	0	0	5 [42]	5
Enforcement/compliance	13 [43–55]	4 [48, 56, 57]	0	17
				23
Effectiveness of policy	17 [9, 58–72]	5 [60, 73–75]	1 [76]	19
				10
Industry misconduct	17 [77–85]	2 [82, 86]	0	74
Other	5 [87–90]	4 [88, 91, 92]	1 [92]	
Total pieces of evidence	52	15	7	

### Raising the age of sale

All evidence about the age-of-sale policies was anecdotal and found in articles that focused on a different topic than agenda setting, policy formulation and/or legitimation (Table 1). Thirty pieces of evidence were found in articles that had enforcement/compliance (*n* = 13) or effectiveness of the policy (*n* = 17) as the main focus. Much of this evidence was from papers on the implementation of the federal Synar Amendment of the United States, in which the authors commented on the adoption of age-of-sale policies by individual states in response to this amendment. Seventeen pieces of evidence about age-of-sale policies were found in articles with a main focus on industry misconduct, in which the authors commented on how the industry promoted voluntary agreements as alternative policy solutions. The authors referred to these agreements as ineffective by design and noted that they were intended to ward off more comprehensive and effective legislation.

### Limiting the number or type of tobacco outlets

Fifteen pieces of evidence were found that concerned limiting the number or type of tobacco outlets. Again, no evidence was found in articles that had agenda setting, policy formulation and/or legitimation as the main focus. Most pieces of evidence focused on the enforcement of and compliance with the policy (*n* = 4) or the effectiveness of the policy (*n* = 5). The pieces of evidence were all anecdotal, meaning that they were not found in the results section of the article and were not based on a systematic analysis of data.

### Banning tobacco displays

We found seven pieces of evidence related to banning tobacco displays. Five of these came from one paper [42]. This was the only article in our database that focused on the first stages of the policy cycle and that offered a systematic analysis of data concerning the policymaking process. These authors examined the adoption of display bans in two Australian jurisdictions. The empirical analysis showed how the ban was legitimized by framing it in terms of youth prevention and combining the ban with other policy measures, thus generating strong public support for these measures. Furthermore, presenting the ban as a natural extension of existing advertising bans increased its attractiveness to policymakers. Evidence was also presented regarding the agenda setting phase, which described how a widely accepted and highly compelling evidence base about tobacco control interventions in general created a favorable political environment. This environment enabled the passage of a tobacco display ban without an explicit prior analysis of scientific evidence in support of the ban [42]. The remaining two pieces of evidence were anecdotal and described the FCTC, endgame strategies, and their agenda setting functions.

### Distribution across time

**Fig. 2:**
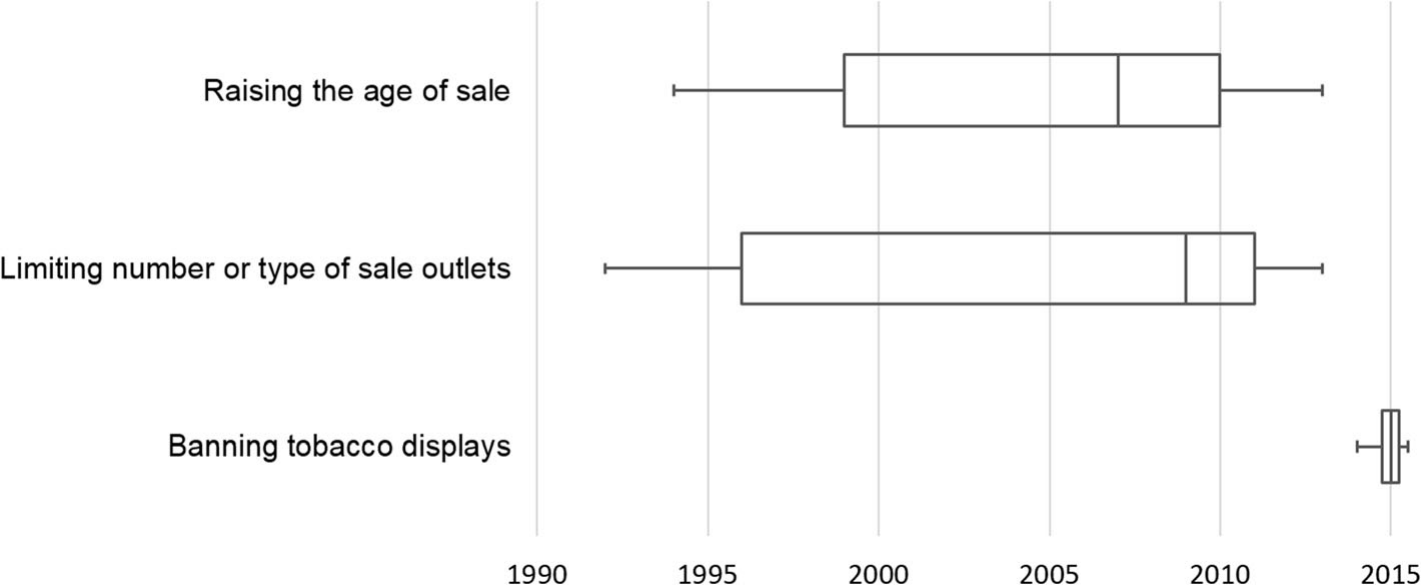
Dispersion of published evidence across time

Figure 2 presents boxplots of the distribution across time of the publication of the identified pieces of evidence regarding the three policy measures. The evidence on the policy process of the two youth access policies was published prior to evidence on the policy process of the display bans.

## Discussion

Our study showed that scientific, systematic evidence on the first stages of the policy process is scarce for the three policies under study. Most evidence was anecdotal, i.e., restricted to incidental observations presented as accounts of the authors. We were able to identify only one study that presented systematic scientific evidence on the policy process [42]. This study provided evidence on the agenda setting and legitimation phases of policy processes that ultimately resulted in the adoption of display bans in two Australian jurisdictions [42].

These findings support the general claim of Clavier & de Leeuw [30] that the policymaking process is understudied in the health promotion field, at least as far as youth prevention policies in the field of tobacco control are concerned. Scholars often study what happens after a policy has been adopted (e.g., the implementation, evaluation and policy maintenance stages). The predominance of this late-stage focus is further illustrated by our finding that most pieces of anecdotal evidence that we found regarding the early phases of the policy process were identified in papers that mainly focused on later stages of the process (e.g., implementation, evaluation and policy maintenance).

Why are the initial phases of the policy process understudied in research on policies to limit youth access and exposure to tobacco? The answer might be that public health scientists consider policymaking to be an abstract construct that is best left to the domain of politics [93]. De Leeuw et al. [93] remark that only a few health promotion scholars are trained in the political sciences. The interest of researchers trained in health promotion or public health may not lie in the “obscure” and hard-to-grasp process of policymaking [93]. Moreover, researchers who are trained in the behavioral or psychological sciences may be more inclined to study the behavior of individuals in response to a certain tobacco control policy. Tobacco control policies may then be merely considered distal determinants of health [93].

In describing the relationship between science and policymaking, Larsen [94] argues that the tobacco control research literature can be classified into two broad categories: a science-driven body and a policy-driven one. Research in the science-driven category often concludes that policymakers should base their policies on scientific findings, which are considered to be immediate and sufficient causes for the formulation of policies. Smith [95] makes a similar claim in the wider domain of public health policy by describing a “knowledge-driven model” in which research findings are assumed to provide the necessary pressure for policy to develop. Politics are then merely seen as a “barrier”, which science must overcome. The second body of tobacco control research, Larsen [94] claims, is smaller in size and regards scientific findings as one among many factors that influence policymakers’ decisions. This category typically focuses on the dynamics and institutional surroundings of public policy [94].

It seems that most literature that we found could be grouped into the first category (i.e., the science- or knowledge-driven body of literature), which is often reflected by normative comments in the discussion sections in which authors conclude that policymakers should consider scientific evidence about effectiveness to base policymaking decisions on. However, without rigorous scientific assessment of the first stages, it remains uncertain how the outcomes of studies on effectiveness, enforcement or compliance could be relevant to policymaking in these initial phases. Whereas advocates stress the importance of evidence in their work and define it as being central to their advocacy, politicians and political advisors may be more inclined to listen to economic and ideological arguments in governmental debates [96].

A possible limitation of this study was that, due to time and resource constraints, the full-text screening of the 200 articles was performed by one individual. Full-text screening by two individuals may have resulted in slightly more or fewer abstracted anecdotal pieces of evidence. However, the main conclusion of our study remains valid: there is only one article that focuses on the first three stages of the policy process.

If we want to understand the substantial variation in tobacco control policy adoption across different countries [97], we need to gain more insight into the first phases of the policy process. Many ideas circulate about what causes this variation in policy adoption; however, there is barely any scientific evidence on why policy processes have led to such different outcomes in different countries. Moreover, a better understanding of such processes may be of crucial importance for tobacco control advocates to work more effectively.

## Conclusion

The processes influencing the adoption of youth access and exposure policies have been grossly understudied. We identified only one study that systematically examined the first stages of the policy process of tobacco display bans in two Australian jurisdictions. Aside from the evidence resulting from this study, which was based on a scientific analysis of data, all other evidence we found was merely anecdotal and restricted to author accounts. We therefore call on researchers to devote more attention to the initial phases of the policy process of youth prevention policies in tobacco control. Specifically, this means systematic empirical research that employs existing theories on the process of policymaking [33–38] and that utilizes, when possible, a comparative approach. A better understanding of these three first phases, as they are relevant to policy adoption, is essential to understand country variations in tobacco control policy and to help tobacco control advocates use the political arena more effectively.

## Additional files

Additional file 1: Search strategies. (DOC 27 kb)

Additional file 2: Extracted evidence. (DOC 191 kb)

## Data Availability

The generated dataset and search strategies are attached to the supplementary file.

## Abbreviations

FCTC: Framework Convention on Tobacco Control
RAMESES: Realist And Meta-narrative Evidence Synthesis: Evolving Standards
SSCI: Social Sciences Citation Index
