# Supplementary material for: Policies that limit youth access and exposure to tobacco: a scientific neglect of the first stages of the policy process
Source: BMC Public Health. 2019 Jun 26;19:825. doi: 10.1186/s12889-019-7073-x (PMC6595563; doi:10.1186/s12889-019-7073-x)
Supplement: Supplementary file 1 — Search strategies. (DOC 27 kb) [file 12889_2019_7073_MOESM1_ESM.doc]

**APPENDIX**

**Search PubMed**

1. "Jurisprudence" OR "legislation" OR "policy" OR "Health Policy"

2. "Smok*" OR "tobacco" OR "youth smoking" [Title/Abstract]

3. "age of sale" OR "Legal purchase" OR"Legal age" OR "minimum age law" OR "minimum legal purchase age" OR "Access intervention*" OR "Youth access" OR "Access to tobacco"

4. "Display ban*" OR "Point of sale tobacco" OR "Retail cigarette advertising" OR "Retail tobacco marketing"

5. "Convenience store concentration" OR "Outlet density" OR "Retail tobacco availability" OR "Retail tobacco outlet density" OR "Retailer density" OR "Tobacco outlet density" OR "Tobacco retail outlet*" OR "Tobacco retailer density"

6. "vending machine*" OR "voluntary agreement*" OR "self-regulation" OR "Self-preservation" OR "Corporate Social Responsibility"

7. "e-cigarette*" [Title] OR "electronic cigarette*" [Title] OR "electronic nicotine delivery systems" [Title] OR "Waterpipe" [Title] OR "public opinion*" [Title] OR "smoking prevalence" [Title] OR "Attitudes" [Title] OR "quit attempt" [Title] OR "quit success" [Title] OR "Simsmoke" [Title]

8. #3 OR #4 OR #5 OR #6

9. #1 AND #2 AND #8

10. #9 NOT #7

**Search Web of Science (SSCI)**1. TS=("Jurisprudence") OR TS=("legisl*") OR TS=("polic* process*") OR TS=("Health Polic*") OR TS=("Policy NEAR/3 mak*")

2. TS=("Smok*") OR TS=("tobacco") OR TI=("youth smoking")

3. TS=("Age of sale") OR TS=("Legal purchase") OR TS=("legal NEAR/3 age") OR TS=("Minimum age law") OR TS=("Minimum legal purchase age") OR TS=("Access intervention*") OR TS=("Youth NEAR/5 access") OR TS=("Access NEAR/5 tobacco") OR TS=("Sale* NEAR/5 minor*") OR TS=("Access law*") OR TS=(display NEAR/5 ban*) OR TS=(tobacco NEAR/5 display*) OR TS=("Convenience store concentration") OR TS=("Outlet density") OR TS=("Retail tobacco outlet density") OR TS=("Retail* density") OR TS=("Tobacco retail* outlet*") OR TS=("vending machine*") OR TS=("voluntary agreement*") OR TS=("self-regulation") OR TS=("Self-preservation") OR TS=("Corporate Social Responsibility")

4. TI=("e-cigarette*") OR TI=("electronic cigarette*") OR TI=("electronic nicotine delivery system*") OR TI=("Waterpipe") OR TI=("public opinion*") OR TI=("smoking prevalence") OR TI=("Attitudes") OR TI=("quit attempt") OR TI=("quit success") OR TI=("Simsmoke") OR TI=("Smokeless")

5. #1 AND #2 AND #3

6. #5 NOT #4
