# Supplementary material for: Policies that limit youth access and exposure to tobacco: a scientific neglect of the first stages of the policy process
Source: BMC Public Health. 2019 Jun 26;19:825. doi: 10.1186/s12889-019-7073-x (PMC6595563; doi:10.1186/s12889-019-7073-x)
Supplement: Supplementary file 2 — Extracted evidence. (DOC 191 kb) [file 12889_2019_7073_MOESM2_ESM.doc]

EXTRACTED EVIDENCE

| **Nr** | **Art. Nr** | **Article title** | **Authors** | **Year** | **Focus of article** | **Stage** | **Outcome** | **Information** |
| --- | --- | --- | --- | --- | --- | --- | --- | --- |
| 1 | 1 | State laws on youth access to tobacco in the United States: measuring their extensiveness with a new rating system. | Alciati, M. H.; Frosh, M.; Green, S. B.; Brownson, R. C.; Fisher, P. H.; Hobart, R.; Roman, A.; Sciandra, R. C.; Shelton, D. M. | 1998 | Effectiveness of policy/policies | Policy Formulation | Age of Sale | An important catalyst for state legislation in 1993 and beyond was the Synar Amendment to the Public Health Service Act of 1992. As a condition for receiving certain block grants, this act requires states to provide evidence of enforcement of state laws setting a minimum age of 18 years for minors’ access to tobacco, and requires states to conduct “random, unannounced inspections” of tobacco sellers. |
| 2 | 2 | The “We Card” program: Tobacco industry “youth smoking prevention” as industry self-preservation. | Apollonio, D. E., & Malone, R. E. | 2010 | Industry misconduct | Policy Formulation | Age of Sale | In short, We Card training efforts helped gain the recognition of hundreds of elected officials on the local, state and federal levels through hundreds of positive news stories. Press reports citing We Card appear in major dailies (USA Today, LA Times), in many local newspapers and on television nationwide. State retail association executives continue to point to We Card training as evidence that further restrictions and taxes aren’t necessary. |
| 3 | 2 | The “We Card” program: Tobacco industry “youth smoking prevention” as industry self-preservation. | Apollonio, D. E., & Malone, R. E. | 2010 | Industry misconduct | Policy Formulation | Age of Sale | However, despite industry claims that the program is effective, internal industry evidence suggests that We Card has not reduced tobacco sales to minors and that it was not designed to do so. Instead, We Card was explicitly structured to improve the industry’s public image and to thwart regulation and law enforcement activity |
| 4 | 2 | The “We Card” program: Tobacco industry “youth smoking prevention” as industry self-preservation. | Apollonio, D. E., & Malone, R. E. | 2010 | Industry misconduct | Policy Formulation | Age of Sale | Despite continued concerns regarding the program’s effectiveness, CRTR concluded that We Card training provided ‘‘evidence that further restrictions and taxes aren’t necessary. |
| 5 | 2 | The “We Card” program: Tobacco industry “youth smoking prevention” as industry self-preservation. | Apollonio, D. E., & Malone, R. E. | 2010 | Industry misconduct | Policy Formulation | Age of Sale | The tobacco industry and retailers anticipated from the program’s inception that We Card could be used to block stronger policies restricting youth access to tobacco. Industry surveys in 1996 found that retailers saw this as an excellent use of the program. |
| 6 | 3 | Minority youth access to tobacco: a neighborhood analysis of underage tobacco sales. | Asumda, F.; Jordan, L. | 2009 | Enforcement/ Compliance | Agenda setting | Age of Sale | The Synar Amendment to the 1992 Federal Alcohol, Drug Abuse and Mental Health Administration Reorganization Act, which requires all states to enact and enforce laws prohibiting the sale of tobacco to minors (under 18), was passed in 1992 as a legal framework to prevent underage smoking. |
| 7 | 4 | A national survey of public support for restrictions on youth access to tobacco. | Bailey, W. J.; Crowe, J. W. | 1994 | Other | Agenda setting | Age of Sale | As a means of reducing this risk, in 1992, the U.S. Congress enacted the so-called “Synar amendment” to the authorization act providing block grant funding to states for alcohol and other drug abuse prevention and treatment programs, requiring stares to enact and enforce laws restricting youth access to tobacco.” |
| 8 | 5 | A grim contradiction: the practice and consequences of corporate social responsibility by British American Tobacco in Malaysia. | Barraclough, S.; Morrow, M. | 2008 | Industry misconduct | Policy Formulation | Age of Sale | BATM has successfully positioned itself, in both symbolic and substantial terms, as a partner in seeking to prevent youth smoking. This role has been strongly legitimized through official Government endorsement. Some years ago, the Deputy Minister of Youth and Sports, Datuk Ong Tee Keat, had indicated that ‘the government welcomed the participation of the tobacco industry’ in solving the problem of under-age smoking, while the Minister of Domestic, Trade and Consumer Affairs, Tan Sri Dato Muhyiddin bin Mohd Yassin, observed that ‘the government is 100% behind this initiative. |
| 9 | 6 | Tobacco control policy and adolescent cigarette smoking status in the United States. | Botello-Harbaum, M. T.; Haynie, D. L.; Iannotti, R. J.; Wang, J.; Gase, L.; Simons-Morton, B. | 2009 | Effectiveness of policy/policies | Policy Formulation | Age of Sale | Notably, the Synar Amendment (Federal Public Law 102-321) stipulates the minimum age for purchase of tobacco products as 18 years and calls for enforcement of this law with random inspections of over-the-counter and vending machine outlets. |
| 10 | 7 | Environmental and policy interventions to control tobacco use and prevent cardiovascular disease. | Brownson, R. C.; Koffman, D. M.; Novotny, T. E.; Hughes, R. G.; Eriksen, M. P. | 1995 | Effectiveness of policy/policies | Policy Formulation | Age of Sale | Preventing youth from smoking may be enhanced by the recently enacted Synar Amendment to the Alcohol, Drug Abuse, and Mental Health Administration Reorganization Act.60,61 This amendment requires that all states enact and enforce a law prohibiting the sale or distribution of tobacco products to minors (persons under age 18) as a condition of receiving full substance abuse and mental health block grant funds. Legislation should include essential components to strengthen penalties and enforcement of youth access laws, including 1. a licensing system similar to that used to control the sale of alcoholic beverages 2. a minimum age of legal purchase at 19 years […] a ban of vending machines to dispense tobacco products. |
| 11 | 7 | Environmental and policy interventions to control tobacco use and prevent cardiovascular disease. | Brownson, R. C.; Koffman, D. M.; Novotny, T. E.; Hughes, R. G.; Eriksen, M. P. | 1995 | Effectiveness of policy/policies | Policy Formulation | Sale Outlets | Preventing youth from smoking may be enhanced by the recently enacted Synar Amendment to the Alcohol, Drug Abuse, and Mental Health Administration Reorganization Act.60,61 This amendment requires that all states enact and enforce a law prohibiting the sale or distribution of tobacco products to minors (persons under age 18) as a condition of receiving full substance abuse and mental health block grant funds. Legislation should include essential components to strengthen penalties and enforcement of youth access laws, including 1. a licensing system similar to that used to control the sale of alcoholic beverages 2. a minimum age of legal purchase at 19 years […]7. a ban of vending machines to dispense tobacco products |
| 12 | 8 | Politics of Evidence: The Communication of Evidence by "Stakeholders' when Advocating for Tobacco Point-of-sale Display Bans in Australia. | Cenko, Clinton; Pulvirenti, Mariastella | 2015 | Adoption of law | Agenda setting | Display Ban | According to interviewees, this body of evidence has made tobacco control an attractive field for political intervention. In this case, the political environment for tobacco control enabled a government announcement to occur without explicit prior analysis of the evidence in support of display bans. |
| 13 | 8 | Politics of Evidence: The Communication of Evidence by "Stakeholders' when Advocating for Tobacco Point-of-sale Display Bans in Australia. | Cenko, Clinton; Pulvirenti, Mariastella | 2015 | Adoption of law | Legitimation | Display Ban | Numerous interviewees, however, acknowledged that the government framed the questions in this survey to emphasize that display bans would protect young people from taking up smoking. In framing the questions this way, they hoped that they would evoke positive support for the ban in the survey responses. They considered that this approach was ‘clever’ and ‘strategic’. |
| 14 | 8 | Politics of Evidence: The Communication of Evidence by "Stakeholders' when Advocating for Tobacco Point-of-sale Display Bans in Australia. | Cenko, Clinton; Pulvirenti, Mariastella | 2015 | Adoption of law | Legitimation | Display Ban | [...] the packaging of this policy idea with other policy initiatives. In Australia, the other regulations that were introduced together with the display bans included bans on smoking in cars and bans on selling tobacco from temporary booths. This was perceived to be a more compelling way to present the tobacco display ban. As a policy package, health lobby and government officers stated that the initiative was not about one new policy but a group of policies or regulations that together should have an effect on smoking prevalence. |
| 15 | 8 | Politics of Evidence: The Communication of Evidence by "Stakeholders' when Advocating for Tobacco Point-of-sale Display Bans in Australia. | Cenko, Clinton; Pulvirenti, Mariastella | 2015 | Adoption of law | Legitimation | Display Ban | If you asked a question like, ‘Do you support imposing regulations on small retailers even at the cost of their profits if the aim is to get cigarettes out of the sight of children?’, it was returning 90% plus supportive. So certainly we were cognisant that it was a political winner as well. [...] strong public support for introducing a particular law could drive its introduction. |
| 16 | 8 | Politics of Evidence: The Communication of Evidence by "Stakeholders' when Advocating for Tobacco Point-of-sale Display Bans in Australia. | Cenko, Clinton; Pulvirenti, Mariastella | 2015 | Adoption of law | Legitimation | Display ban | The interviews also revealed that often a policy concept, such as tobacco display bans, can be perceived as an evidence-based approach when it is perceived as an extension of another policy concept that does have a strong evidence base. In this instance, the display bans are widely seen as a natural progression from tobacco advertising bans, which are held in high regard in the tobacco control community and in society generally. Consequently, the perception that this was a policy idea that logically flowed on from a related and successful policy appeared to increase its attraction to regulators. |
| 17 | 9 | Youth access to cigarettes in six sub-Saharan African countries. | Chandora, R.; Song, Y.; Chaussard, M.; Palipudi, K. M.; Lee, K. A.; Ramanandraibe, N.; Asma, S. | 2016 | Enforcement/ Compliance | Policy formulation | Age of Sale | Uganda recently introduced a comprehensive tobacco control law in line with many of the recommendations outlined in the WHO FCTC (Anon., 2016). The bill outlines stringent regulations on the distribution, sale, and use of tobacco products, and it specifically prohibits the supply and sale of tobacco products to and by minors (Anon., 2014). |
| 18 | 10 | A comprehensive review of state laws governing Internet and other delivery sales of cigarettes in the United States. | Chriqui, J. F.; Ribisl, K. M.; Wallace, R. M.; Williams, R. S.; O'Connor, J. C.; el Arculli, R. | 2008 | Other | Agenda setting | Sale Outlets | The U.S. Congress held hearings on the problems posed by Internet and mail-order tobacco sales in 2003, and all testifying agreed that something needed to be done to prevent minors’ access to tobacco products via Internet sales and retailer evasion of state taxes […] Thirty-two states’ laws (94% of the states with laws) specifically addressed youth access issues (excluding penalty/ enforcement provisions) […] Although there are no federal laws banning Internet and mail-order tobacco sales, 34 states have some type of law designed to prevent tax evasion, youth access to cigarettes from delivery sales, or both. Five states completely banned delivery sales during our study period ending December 31, 2006 |
| 19 | 11 | The Illegal Sale of Cigarettes to United-States Minors - Estimates by State. | Cummings, K. M.; Pechacek, T.; Shopland, D. | 1994 | Enforcement/ Compliance | Policy formulation | Age of Sale | The Synar Amendment […] provides that, as a condition of receiving block grant funds for substance abuse prevention and treatment, states must enact and enforce a law prohibiting the sale or distribution of tobacco products to individuals under 18 years of age. Specifically, states are required to check retailer compliance with the tobacco age sale law by conducting random, unannounced inspections of locations at which tobacco products are sold and to submit a report to the secretary of health and human services describing their activities aimed at enforcing the law during the preceding year. If a state fails to demonstrate a reasonable effort to enforce the law preventing tobacco sales to minors, then it will lose between 10% and 40% (depending on the year) of its ADAMHAfunds. ADAMHAblock grant awards tentatively set for fiscal year 1993 range from $2.0 million for Wyoming to $201.2 million for California. |
| 20 | 12 | Youth access to tobacco: the effects of age, gender, vending machine locks, and "it's the law" programs. | DiFranza, J. R.; Savageau, J. A.; Aisquith, B. F. | 1996 | Industry misconduct | Policy Formulation | Sale Outlets | Vending machine owners have proposed electronic remote control lockout devices as an alternative to a ban. |
| 21 | 13 | Tobacco control: a brief review of its history and prospects for the future. | Emmons, K. M.; Kawachi, I.; Barclay, G. | 1997 | Other | Policy Formulation | Age of Sale | The recent Synar Amendment to the Alcohol, Drug Abuse, and Mental Health Administration Reorganization now requires all states to enact and enforce a law prohibiting the sale or distribution of tobacco products to minors. The legislation must contain several components, including a licensing system to control sale of the product, penalties for illegal sales to minors, and elimination of the sale of single cigarettes and free distribution of tobacco products. This amendment is particularly important in light of evidence that minors are able to successfully purchase tobacco from stores and vending machines 70% to 100% of the time. |
| 22 | 13 | Tobacco control: a brief review of its history and prospects for the future. | Emmons, K. M.; Kawachi, I.; Barclay, G. | 1997 | Other | Policy Formulation | Age of Sale | The tobacco industry has lobbied extensively for voluntary enforcement strategies related to youth access to tobacco. […] Finally, the industry has developed its own access campaign that is a key part of its strategy to avoid state and federal regulation regarding youth access. |
| 23 | 13 | Tobacco control: a brief review of its history and prospects for the future. | Emmons, K. M.; Kawachi, I.; Barclay, G. | 1997 | Other | Policy Formulation | Sale Outlets | The tobacco industry has responded [to cities that adopted bans and restrictions on vending machines] by proposing alternative policies that are less restrictive than vending machine bans. These proposals include policies to require locking devices on cigarette vending machines that prevent purchase unless the lock is deactivated by an employee. |
| 24 | 14 | Youth access interventions do not affect youth smoking. | Fichtenberg, C. M.; Glantz, S. A. | 2002 | Effectiveness of policy/policies | Policy Formulation | Age of Sale | Youth access has also become a cornerstone of federal tobacco control policy, as reflected in the Synar Amendment to the 1992 Alcohol, Drug Abuse and Mental Health Administration Reorganization Act (Pub L No. 103-312) which required states to enact and enforce a minimum age-of-sale law of 18 years. |
| 25 | 15 | Locking Devices on Cigarette Vending Machines - Evaluation of a City Ordinance. | Forster, J. L.; Hourigan, M. E.; Kelder, S. | 1992 | Enforcement/ Compliance | Policy Formulation | Sale Outlets | Other cities and states are following Minnesota's lead in restricting youth access to tobacco through restrictions on vending machines and other policies. In response, the tobacco and vending machine industries have proposed alternative policies that are less restrictive than vending machine bans, such as requiring cigarette vending machines to be retrofitted with locking devices that prevent cigarette purchase unless the lock is deactivated by an employee. |
| 26 | 15 | Locking Devices on Cigarette Vending Machines - Evaluation of a City Ordinance. | Forster, J. L.; Hourigan, M. E.; Kelder, S. | 1992 | Enforcement/ Compliance | Policy Formulation | Sale Outlets | The only long-term experience with vending machine locking devices comes from Utah, which had a state law until 1989 requiring their installation. The experience in Utah was that the devices were rarely installed (1 of 22 machines was equipped with the device in one informal study), and, where installed, they were rarely operating (B. Neiger, written communication, February 1990). The Utah law was changed in 1989 to prohibit vending machine sales of cigarettes in most locations. |
| 27 | 16 | The effects of community policies to reduce youth access to tobacco. | Forster, J. L.; Murray, D. M.; Wolfson, M.; Blaine, T. M.; Wagenaar, A. C.; Hennrikus, D. J. | 1998 | Effectiveness of policy/policies | Policy Formulation | Age of Sale | These assumptions led the US Department of Health and Human Services, in January 1996, to issue rules to implement the Synar amendment, which requires that each state receiving a federal Substance Abuse Prevention and Treatment Block Grant adopt and enforce a tobacco age-of-sale law and show progressive reductions in tobacco sales to minors. |
| 28 | 17 | Policy interventions and surveillance as strategies to prevent tobacco use in adolescents and young adults. | Forster, J. L.; Widome, R.; Bernat, D. H. | 2007 | Effectiveness of policy/policies | Policy Formulation | Age of Sale | With the hope that effectively limiting the supply of cigarettes to youth would lead to lower cigarette use, Congress mandated in the Synar Amendment that all states and territories must enact laws that prohibit the sale of tobacco to minors and enforce these laws with compliance checks by underage decoys. |
| 29 | 18 | Youth access to tobacco: policies and politics. | Forster, J. L.; Wolfson, M. | 1998 | Effectiveness of policy/policies | Agenda setting | Age of Sale | Downey & Gardiner (43) surveyed representatives of state government agencies and nonprofit tobacco control organizations in each state in 1995 (i.e. subsequent to passage of the Synar legislation and publication of the Notice of Proposed Rule-Making but prior to publication of the final rule in early 1996). The law and proposed rule appear (based on the reports of respondents to the survey) to have been the catalyst for concrete actions in several states, even before publication of the final rule: passage of new vending machine restrictions and increased penalties for age-of-sale violations in Washington, an increase of the minimum age-of-sale to 18 in Georgia, enactment of a licensing law in Maine, and enactment of the comprehensive Stop Tobacco Access to Kids Enforcement (STAKE) Act in California (43). |
| 30 | 18 | Youth access to tobacco: policies and politics. | Forster, J. L.; Wolfson, M. | 1998 | Effectiveness of policy/policies | Policy Formulation | Age of Sale | The “It’s the Law” campaign, introduced by the Tobacco Institute in 1990, included a series of decals, buttons, and educational materials for retailers (122). The primary message was that it is illegal for minors to purchase tobacco, and the intent was to discourage youth from trying to purchase. The pressure felt by retailers and the tobacco industry as the intent to regulate was announced by the FDA and the Synar regulations were being developed was demonstrated by new initiatives that were more comprehensive but still voluntary in nature. The “We Card” campaign, launched by the National Coalition for Responsible Tobacco Retailing, included age calendars, employee training videos, and mystery shopper programs. |
| 31 | 19 | Does adolescent perception of difficulty in getting cigarettes deter experimentation? | Gilpin, E. A.; Lee, L.; Pierce, J. P. | 2004 | Enforcement/ Compliance | Agenda setting | Age of Sale | In compliance with the Synar Amendment, California’s Legislature enacted the Stop Tobacco Access to Kids Enforcement (STAKE) Act in September 1994, which was designed to strengthen enforcement of the existing law banning tobacco sales to minors. The California Department of Health Services was designated to enforce the STAKE Act, which was implemented in January 1995. As part of the enforcement and to comply with the Synar Amendment, it conducted random compliance checks or ‘stings’ beginning in December of 1995. In 1996, the fines and terms of permit revocation for merchants who sold to minors were increased. |
| 32 | 20 | State legislators' beliefs about legislation that restricts youth access to tobacco products. | Gottlieb, N. H.; Goldstein, A. O.; Flynn, B. S.; Cohen, E. J.; Bauman, K. E.; Solomon, L. J.; Munger, M. C.; Dana, G. S.; McMorris, L. E. | 2003 | Other | Agenda setting | Age of Sale | Noncompliance with the Synar Amendment resulted in potential monetary penalties through loss of federal block grant money for substance abuse programs.16 Consequently, many state legislatures were interested in strengthening and enforcing their legislation preventing youth access to tobacco. |
| 33 | 21 | Middle school students' sources of acquiring cigarettes and requests for proof of age. | Gratias, E. J.; Krowchuk, D. P.; Lawless, M. R.; Durant, R. H. | 1999 | Enforcement/ Compliance | Policy formulation | Age of Sale | The 1992 Synar Amendment to the Public Health Act requires states to enact legislation prohibiting the sales and distribution of tobacco products to youths under the age of 18 years in order to continue receiving Federal funds for substance abuse prevention and treatment (6). As a result, all 50 states and the District of Columbia have established the minimum age of purchase for tobacco products at 18 years. |
| 34 | 22 | Long-term effects of laws governing youth access to tobacco. | Grucza, R. A.; Plunk, A. D.; Hipp, P. R.; Cavazos-Rehg, P.; Krauss, M. J.; Brownson, R. C.; Bierut, L. J. | 2013 | Enforcement/ Compliance | Policy Formulation | Age of Sale | Adoption of these initiatives was accelerated by passage of the 1992 Synar Amendment to the Federal Alcohol, Drug Abuse, and Mental Health Administration Reorganization Act, which mandated withholding federal block-grant money from states that failed to prohibit distribution of tobacco products to persons younger than 18 years, or failed to enforce such prohibitions. |
| 35 | 22 | Long-term effects of laws governing youth access to tobacco. | Grucza, R. A.; Plunk, A. D.; Hipp, P. R.; Cavazos-Rehg, P.; Krauss, M. J.; Brownson, R. C.; Bierut, L. J. | 2013 | Enforcement/ Compliance | Policy Formulation | Sale Outlets | Various measures were implemented by individual states to increase retailer compliance with de jure purchase ages, such as restricting physical access to cigarette vending machines and banning the sale of single cigarettes. |
| 36 | 23 | Smoking habits before and after the introduction of a minimum-age law for tobacco purchase: analysis of data on adolescents from three regions of Sweden. | Hagquist, C.; Sundh, M.; Eriksson, C. | 2007 | Effectiveness of policy/policies | Policy Formulation | Age of Sale | Minimum age laws for purchase of tobacco constitute a mandatory part of the WHO convention on tobacco introduced in 2003, which became international law in 2005. |
| 37 | 24 | The implementation and enforcement of tobacco control laws: Policy implications for activists and the industry. | Jacobson, P. D.; Wasserman, J. | 1999 | Enforcement/ Compliance | Agenda setting | Age of Sale | The state laws designed specifically to enforce youth access restrictions to tobacco products in our sample were usually enacted in response to the Synar Amendment. |
| 38 | 25 | Age verification cards fail to fully prevent minors from accessing tobacco products. | Kanda, H.; Osaki, Y.; Ohida, T.; Kaneita, Y.; Munezawa, T. | 2011 | Effectiveness of policy/policies | Policy Formulation | Sale Outlets | Countries that have ratified the Framework Convention on Tobacco Control (FCTC) must legally prohibit the introduction of tobacco vending machines to minors, and when appropriate, conduct a total ban of tobacco vending machines. |
| 39 | 26 | Tobacco retail outlet advertising practices and proximity to schools, parks and public housing affect Synar underage sales violations in Washington, DC. | Kirchner, T. R.; Villanti, A. C.; Cantrell, J.; Anesetti-Rothermel, A.; Ganz, O.; Conway, K. P.; Vallone, D. M.; Abrams, D. B. | 2015 | Enforcement/ Compliance | Policy Formulation | Age of Sale | The US Congress passed the Synar amendment in 1992, providing funding from the Substance Abuse and Mental Health Services Administration (SAHMSA) to states to enact and enforce a prohibition on the sale of tobacco to minors. The regulation requires that all 50 states and nine jurisdictions […] enforce a youth tobacco sales prohibition via unannounced inspections across a representative sample of tobacco outlets, and that they maintain an annual non-compliance rate of less than 20%. |
| 40 | 27 | Validity of assessments of youth access to tobacco: the familiarity effect. | Landrine, H.; Klonoff, E. A. | 2003 | Enforcement/ Compliance | Policy Formulation | Age of Sale | Foremost among policy-level efforts to reduce youth access to tobacco are the federal *Synar Amendment*1,2 and the subsequent state3,4 and local5 implementations of the amendment. |
| 41 | 28 | The vector of the tobacco epidemic: tobacco industry practices in low and middle-income countries. | Lee, S. Ling, P. M. Glantz, S. A. | 2012 | Industry misconduct | Policy Formulation | Age of Sale | On the surface, the industry promoted programs to discourage youth smoking, however, the actual purpose of the tactic was to advertise the tobacco industry themselves as a responsible corporate, in turn to undermine effective tobacco-control interventions. |
| 42 | 28 | The vector of the tobacco epidemic: tobacco industry practices in low and middle-income countries. | Lee, S. Ling, P. M. Glantz, S. A. | 2012 | Industry misconduct | Policy Formulation | Age of Sale | Youth smoking prevention programs have long been used as part of the tobacco industry’s CSR programs to argue that government programs to reduce tobacco use are unnecessary and to appeal that the industry makes efforts to prevent children from smoking. |
| 43 | 29 | Expert opinions on optimal enforcement of minimum purchase age laws for tobacco. | Levy, D. T.; Chaloupka, F.; Slater, S. | 2000 | Enforcement/ Compliance | Policy Formulation | Age of Sale | In response to the easy access youth had to tobacco products, the federal government passed the Synar Amendment (PL 103-312, Section 1926) in July 1992. To avoid losing federal funds, states must enact laws prohibiting the sale of tobacco to youth […] |
| 44 | 30 | Prices, policies and youth smoking, May 2001. | Liang, L.; Chaloupka, F.; Nichter, M.; Clayton, R. | 2003 | Effectiveness of policy/policies | Policy Formulation | Age of Sale | In 1992, Congress mandated that by the fiscal year of 1994, every state and territory must legally prohibit the sale of tobacco to minors (USDHHS 1995). This congressional mandate, usually referred to as the Synar Amendment, also requires the states to enforce the law by conducting random unannounced checks or inspections every year. |
| 45 | 31 | Tobacco use among school-going adolescents (11-17 years) in Ghana. | Mamudu, H. M.; Veeranki, S. P.; John, R. M. | 2013 | Other | Policy Formulation | Age of Sale | The bill was unanimously passed by the Parliament on July 11, 2012, to bring the country into compliance with the WHO Framework Convention on Tobacco Control (FCTC), the first international public health treaty negotiated by the WHO to deal with the use and spread of tobacco around the world (WHO, 2003), which Ghana ratified in November 2004. |
| 46 | 32 | Increasing the age for the legal purchase of tobacco in England: impacts on socio-economic disparities in youth smoking. | Millett, C.; Lee, J. T.; Gibbons, D. C.; Glantz, S. A. | 2011 | Effectiveness of policy/policies | Policy Formulation | Age of Sale | Consistent with the WHO Framework Convention for Tobacco Control19 a number of European countries, including the UK, France, Germany and Denmark have joined numerous other countries in raising the age for the legal purchase of tobacco to 18 years in recent years. |
| 47 | 33 | Profits and pandemics: prevention of harmful effects of tobacco, alcohol, and ultra-processed food and drink industries. | Moodie, R.; Stuckler, D.; Monteiro, C.; Sheron, N.; Neal, B.; Thamarangsi, T.; Lincoln, P.; Casswell, S. | 2013 | Industry misconduct | Policy Formulation | Age of Sale | Industry documents released because of tobacco46 and asbestos20 litigation show how these industries affect public health legislation and avoid regulation with both hard power (i.e., building financial and institutional relations) and soft power (i.e., influence of culture, ideas, and cognitions of people, advocates, and scientists). |
| 48 | 33 | Profits and pandemics: prevention of harmful effects of tobacco, alcohol, and ultra-processed food and drink industries. | Moodie, R.; Stuckler, D.; Monteiro, C.; Sheron, N.; Neal, B.; Thamarangsi, T.; Lincoln, P.; Casswell, S. | 2013 | Industry misconduct | Policy Formulation | Age of Sale | Furthermore, legislation for clean air,105 asbestos,106 road trauma,30 and tobacco86 was introduced only after the repeated failures of the industries responsible for solving these problems through self-regulation. |
| 49 | 34 | Have Tobacco 21 Laws Come of Age? | Morain, S. R.; Winickoff, J. P.; Mello, M. M. | 2016 | Effectiveness of policy/policies | Legitimation | Age of Sale | Among the interest groups best placed to counteract industry opposition are medical and health professional organizations. The American Medical Association, the American Academy of Pediatrics, the American Academy of Family Physicians, and the American Public Health Association all publicly support Tobacco 21 laws. Active engagement by these and other organizations is critical to ensuring that the policy frame for these laws remains focused on the health benefits, despite efforts to recast the laws as anti–small-business measures. |
| 50 | 35 | The merchants, not the customers: resisting the alcohol and tobacco industries' strategy to blame young people for illegal alcohol and tobacco sales. | Mosher, J. F. | 1995 | Industry misconduct | Policy Formulation | Age of Sale | This legislation requires that states make it illegal to sell tobacco to those under 18 years of age, and that they adopt enforcement procedures for insuring that the illegal-sales laws are obeyed. States which do not comply with the legislation and the implementing regulations face reductions in federal block grant funds. |
| 51 | 36 | Implementation and research priorities for FCTC Articles 13 and 16: tobacco advertising, promotion, and sponsorship and sales to and by minors. | Nagler, R. H.; Viswanath, K. | 2013 | Industry misconduct | Policy Formulation | Sale Outlets | Supported by this evidence base, the FCTC issued several recommendations to limit minor access to tobacco. […] Consider prohibiting the introduction of vending machines in the Party’s jurisdiction, or a total ban on vending machines. |
| 52 | 36 | Implementation and research priorities for FCTC Articles 13 and 16: tobacco advertising, promotion, and sponsorship and sales to and by minors. | Nagler, R. H.; Viswanath, K. | 2013 | Industry misconduct | Policy Formulation | Age of Sale | Although companies claim that they oppose the sale of tobacco to minors, research has shown that they have worked to prevent the enforcement of underage sales laws. One strategy they have used is preempting stronger legislation by advocating self-regulation. |
| 53 | 37 | Effectiveness of state and federal government agreements with major credit card and shipping companies to block illegal Internet cigarette sales. | Ribisl, K. M.; Williams, R. S.; Gizlice, Z.; Herring, A. H. | 2011 | Effectiveness of policy/policies | Agenda setting | Sale Outlets | Because of multiple violations of state and federal laws governing taxation and sales to minors, the U.S. Bureau of Alcohol, Tobacco, Firearms and Explosives and several state Attorneys General (AGs) reached a landmark voluntary agreement on March 17, 2005 with the major credit card companies and PayPal to ban the processing of credit card payments for ICVs. |
| 54 | 38 | The effectiveness of tobacco sales ban to minors: the case of Finland. | Rimpela, A. H.; Rainio, S. U. | 2004 | Effectiveness of policy/policies | Agenda setting | Age of Sale | An increase in adolescents’ smoking at the end of the 1980s led to a critical discussion on the success of anti-smoking policies and in this context also the implementation of the sales ban was assessed. |
| 55 | 39 | Point-of-sale tobacco promotion and youth smoking: a meta-analysis. | Robertson, L.; Cameron, C.; McGee, R.; Marsh, L.; Hoek, J. | 2016 | Effectiveness of policy/policies | Policy Formulation | Display Ban | As defined by the WHO Framework Convention on Tobacco Control, a ‘comprehensive’ ban on tobacco promotion includes the POS, since these are essentially forms of advertising. |
| 56 | 40 | Implementation of electronic locking devices for adolescents at German tobacco vending machines: intended and unintended changes of supply and demand. | Schneider, S.; Meyer, C.; Yamamoto, S.; Solle, D. | 2009 | Effectiveness of policy/policies | Policy Formulation | Sale Outlets | As early as 1997, the German Association of Tobacco Wholesalers and Vending Machine Distributors agreed to a ‘‘voluntary self-restriction’’ proposed by the German Federal Ministry of Health. This restriction prevented the installation of vending machines and tobacco advertisements within 50 and 100 m, respectively, of schools and youth centers. Unfortunately, this has done little to affect access to cigarettes by those under 16 years.7 Similarly, the introduction of the electronic devices is largely considered by national smoking prevention experts to be another concession by the tobacco industry to avoid or delay a complete ban on tobacco vending machines. |
| 57 | 40 | Implementation of electronic locking devices for adolescents at German tobacco vending machines: intended and unintended changes of supply and demand. | Schneider, S.; Meyer, C.; Yamamoto, S.; Solle, D. | 2009 | Effectiveness of policy/policies | Agenda setting | Sale Outlets | Article 16 advocates for two basic options concerning tobacco vending machines: limit access for adolescents or enact a total ban on tobacco vending machines. Germany opted for the first alternative, thus on January 1, 2007, a change in the Youth Protection Law (Jugendschutzgesetz (Article 1055 JuSchG)) was introduced. This law stipulates the mandatory installation of electronic security devices on all vending machines in Germany. With the electronic security device, consumers are required to insert some form of electronic identification (eg, electronic cash card or a European driving licence) to purchase cigarettes from vending machines. |
| 58 | 41 | What Happens After the Implementation of Electronic Locking Devices for Adolescents at Cigarette Vending Machines? A Natural Longitudinal Experiment From 2005 to 2009 in Germany. | Schneider, Sven; Gruber, Johannes; Yamamoto, Shelby; Weidmann, Christian | 2011 | Other | Policy Formulation | Sale Outlets | Article 16 of the “WHO Framework Convention on Tobacco Control” (WHO, 2003), signed by the Federal Republic of Germany in 2003 and ratified in 2004, proposes two basic options concerning tobacco vending machines: access limita­tion for adolescents or a total ban on tobacco vending machines. |
| 59 | 41 | What Happens After the Implementation of Electronic Locking Devices for Adolescents at Cigarette Vending Machines? A Natural Longitudinal Experiment From 2005 to 2009 in Germany. | Schneider, Sven; Gruber, Johannes; Yamamoto, Shelby; Weidmann, Christian | 2011 | Other | Agenda setting | Sale Outlets | In 2007 and 2009, a change in the Youth Protection Law (Jugendschutzgesetz: JuSchG§§10) was introduced to tackle this [unhindered and anonymous access] problem. This law prohibits the sale of cigarettes to minors and enforces the installation of electronic security devices on all cigarette vending machines in Germany. |
| 60 | 42 | Attempts to undermine tobacco control: tobacco industry "youth smoking prevention" programs to undermine meaningful tobacco control in Latin America. | Sebrie, E. M.; Glantz, S. A. | 2007 | Industry misconduct | Policy Formulation | Age of Sale | The tobacco industry has been successful in partnering with third-party allies in Latin America. In addition to allies in the business community, such as tobacco retailers and the hospitality industry,68 the tobacco industry identified a local nonprofit educational organization in each country to recruit its education programs. […] These efforts helped portray the tobacco companies as concerned corporate citizens69 and have created an opportunity in which some politicians (e.g., in Ecuador) allowed the tobacco industry to draft and water down “anti-tobacco” legislation. The most important outcome that tobacco companies achieved in several countries was the endorsement by public officials and national authorities, in particular the education and health ministries. This tactic both legitimizes the tobacco industry and helps it build ties with government that could be valuable in opposing future tobacco control policies. |
| 61 | 42 | Attempts to undermine tobacco control: tobacco industry "youth smoking prevention" programs to undermine meaningful tobacco control in Latin America. | Sebrie, E. M.; Glantz, S. A. | 2007 | Industry misconduct | Policy Formulation | Age of Sale | Beginning in the United States in the early 1980s, the tobacco industry has promoted 4 types of “youth smoking prevention” programs to avoid effective tobacco control policies6: programs directed at parents (e.g., the Tobacco Institute’s “Helping Youth Decide,” 1984), youths (e.g., RJR’s “Right Decisions, Right Now,” 1991), and retailers (e.g., Philip Morris USA’s “Action Against Access,” 1995) and programs providing funding to youth organizations (e.g., 4-H’s “Health Rocks”). During the 1980s and 1990s, the industry spread these programs to the United Kingdom, Canada, Japan,6 Australia,7 Malaysia,8 Singapore,9 Eastern Europe,6 Bangladesh,10 and Argentina. |
| 62 | 42 | Attempts to undermine tobacco control: tobacco industry "youth smoking prevention" programs to undermine meaningful tobacco control in Latin America. | Sebrie, E. M.; Glantz, S. A. | 2007 | Industry misconduct | Policy Formulation | Age of Sale | From April 18 to 21, 1995, Leiber participated in a session on “youth initiatives” at a PMLA Corporate Affairs workshop in Washington, DC. The participants identified a set of required elements for any youth program: • Need for a [voluntary industry] Marketing Code. • Self-regulation as the basis for law and enforcement. “Be ahead of the government.” • Commitment of PM to an initiative and willingness to do it alone; if the industry is not interested. • Aggressively communicate to appropriate audiences what is being done. • Gain government endorsement/ involvement. • Use allies (i.e., retailers).24 […]job, we realize that if we are unable to develop a system for enforcement, *we will continue to be subjected to the threats of the anti-tobacco movement and the restrictive legislation which results* [emphasis added]. |
| 63 | 43 | The food industry and self-regulation: standards to promote success and to avoid public health failures. | Sharma, L. L.; Teret, S. P.; Brownell, K. D. | 2010 | Industry misconduct | Policy Formulation | Age of Sale | The tobacco industry’s development of youth smoking prevention campaigns is arguably 1 of the most extreme examples of an industry abusing self-regulation to deflect legislative action. In response to public and government outcries over marketing to youths, the industry developed several youth smoking prevention programs in the early 1980s.41 These included youth access initiatives (e.g., the Coalition for Responsible Tobacco Retailing’s WeCare, the Tobacco Institute’s It’s the Law, and Philip Morris’s Action Against Access), sponsored educational programs (e.g., the Tobacco Institute’s COURSE Consortium and RJ Reynolds’s Right Decisions Right Now), youth program partnerships (e.g., with the US Junior Chamber of Commerce and the National 4-H Council), and media campaigns (e.g., Philip Morris’s Think. Don’t Smoke.) |
| 64 | 43 | The food industry and self-regulation: standards to promote success and to avoid public health failures. | Sharma, L. L.; Teret, S. P.; Brownell, K. D. | 2010 | Industry misconduct | Policy Formulation | Age of Sale | The tobacco industry’s self-regulatory tactics illustrate the central danger of self-regulation: an industry can use programs and approaches that appear credible and are framed as in the public’s interest but prevent legislation or regulation and damage public health. |
| 65 | 44 | State laws on tobacco control--United States, 1995. | Shelton, D. M.; Alciati, M. H.; Chang, M. M.; Fishman, J. A.; Fues, L. A.; Michaels, J.; Bazile, R. J.; Bridgers, J. C., Jr.; Rosenthal, J. L.; Kutty, L.; et al., | 1995 | Effectiveness of policy/policies | Policy Formulation | Age of Sale | In July 1992, Congress enacted Section 1926 of the Public Health Service Act (the Synar Amendment), which requires states to enact legislation restricting the sale and distribution of tobacco products to minors as a condition of receiving Federal substance abuse prevention and treatment block grant funds. Under this provision, states are also required to enforce these laws in a manner "that can reasonably be expected to reduce the extent to which tobacco products are available to individuals under the age of 18". |
| 66 | 45 | Prohibiting juvenile access to tobacco: Violation rates, cigarette sales, and youth smoking. | Spivak, A. L.; Monnat, S. M. | 2015 | Effectiveness of policy/policies | Policy Formulation | Age of Sale | The authors noted that the tobacco industry’s legislative activities surrounding the program included lobbying efforts to preempt existing tobacco control efforts and even to prohibit official Synar-style compliance operations. |
| 67 | 46 | Compliance with a minimum-age law of 18 for the purchase of tobacco - the case of Sweden. | Sundh, M.; Hagquist, C. | 2006 | Enforcement/ Compliance | Agenda setting | Age of Sale | In the 1990s, the demand for restrictions on tobacco sale increased significantly in Sweden and in 1996 two large supermarket chains introduced a voluntary age limit of 18. The following January, the age limit of 18 became law, covering both cigarettes and smokeless tobacco. |
| 68 | 47 | Differential impact of state tobacco control policies among race and ethnic groups. | Tauras, J. A. | 2007 | Effectiveness of policy/policies | Policy Formulation | Age of Sale | The Synar Amendment, passed by Congress in 1992, put into place a national youth-access policy. It specifies that states lose Federal funding for mental health programs if they fail to adopt and enforce stringent regulations adequately. As part of Synar, states are required to set the minimum age for the legal purchase of tobacco products at 18 or higher. |
| 69 | 48 | Effect of local youth-access regulations on progression to established smoking among youths in Massachusetts. | Thomson, C. C.; Hamilton, W. L.; Siegel, M. B.; Biener, L.; Rigotti, N. A. | 2007 | Effectiveness of policy/policies | Policy Formulation | Age of Sale | In the US, the federal Synar amendment has, since 1996, required all states to have and enforce youth-access laws and document a specific level of compliance or risk loss of federal block grant funds. Many US communities have gone beyond state laws and adopted local ordinances with stronger youth-access provisions. |
| 70 | 49 | "Stay away from them until you're old enough to make a decision": tobacco company testimony about youth smoking initiation. | Wakefield, M.; McLeod, K.; Perry, C. L. | 2006 | Industry misconduct | Policy Formulation | Age of Sale | Engagement in efforts to ostensibly prevent sale of cigarettes to minors has allowed the tobacco industry to develop strategic links with retailers and legislators, facilitating effective lobbying against the implementation of youth access laws and laws prohibiting sales to minors at local, state and federal levels and also weakening point of purchase legislation. |
| 71 | 50 | Store tobacco policies: a survey of store managers, California, 1996-1997. | Weinbaum, Z.; Quinn, V.; Rogers, T.; Roeseler, A. | 1999 | Enforcement/ Compliance | Agenda setting | Age of Sale | The federal Synar Amendment (PHS Act Section No 1926) requires states receiving funds for prevention and treatment of substance abuse to enforce laws to reduce illegal sales of tobacco to minors. In response to the Synar Amendment, California enacted the Stop Tobacco Access to Kids Enforcement (STAKE) Act in 1994, and started to enforce the law in December 1995. |
| 72 | 51 | Facilitating adolescent smoking: who provides the cigarettes? | White, M. M.; Gilpin, E. A.; Emery, S. L.; Pierce, J. P. | 2005 | Enforcement/ Compliance | Policy Formulation | Age of Sale | The Synar Amendment, passed by Congress in 1992, required each state to have and enforce an effective law that set the minimum age of cigarette purchase at 18 years of age; monitoring of state compliance included random, unannounced inspections of venues selling tobacco to determine the rate of selling tobacco to underage buyers, with states required to maintain an illegal sales rate of below 20%. |
| 73 | 52 | Advancing the retail endgame: public perceptions of retail policy interventions. | Whyte, Gregor; Gendall, Philip; Hoek, Janet | 2014 | Other | Agenda setting | Display Ban | However, the rapid reductions in smoking prevalence required to achieve ‘endgame goals’ set by New Zealand and other countries, behove consideration of additional measures, particularly, given retail outlets remain permissive tobacco marketing environments. |
| 74 | 53 | Internet cigarette vendor compliance with credit card payment and shipping bans. | Williams, R. S.; Ribisl, K. M. | 2014 | Enforcement/ Compliance | Agenda setting | Sale Outlets | Because most Internet cigarette vendors (ICVs) were clearly violating laws related to tax reporting and sales to minors, the federal government pursued landmark voluntary agreements in 2005 with the major credit card companies (Office of the New York State Attorney General, 2005), PayPal (Office of the New York State Attorney General, 2005), United Parcel Service (UPS) (Attorney General of the State of New York Health Care Bureau, 2005b), DHL (Attorney General of the State of New York Health Care Bureau, 2005a), and Federal Express (FedEx) (Federal Express, 2003) to prohibit the processing of credit card payments and shipping of cigarettes for ICVs (Office of the New York State Attorney General, 2005). |

1. Alciati MH, Frosh M, Green SB, Brownson RC, Fisher PH, Hobart R, et al. State laws on youth access to tobacco in the United States: measuring their extensiveness with a new rating system. Tob Control. 1998;7:345–52.

2. Apollonio DE, Malone RE. The “We Card” program: tobacco industry “youth smoking prevention” as industry self-preservation. Am J Public Health. 2010; doi:10.2105/AJPH.2009.169573.

3. Asumda F, Jordan L. Minority youth access to tobacco: a neighborhood analysis of underage tobacco sales. Health Place. England; 2009; doi:10.1016/j.healthplace.2008.03.006.

4. Bailey WJ, Crowe JW. A national survey of public support for restrictions on youth access to tobacco. J Sch Health. 1994; doi:10.1111/j.1746-1561.1994.tb03318.x.

5. Barraclough S, Morrow M. A grim contradiction: the practice and consequences of corporate social responsibility by British American Tobacco in Malaysia. Soc Sci Med. 2008; doi:10.1016/j.socscimed.2008.01.001.6.

6. Botello-Harbaum MT, Haynie DL, Iannotti RJ, Wang J, Gase L, Simons-Morton B. Tobacco control policy and adolescent cigarette smoking status in the United States. Nicotine Tob Res. 2009; doi:10.1093/ntr/ntp081.

7. Brownson RC, Koffman DM, Novotny TE, Hughes RG, Eriksen MP. Environmental and policy interventions to control tobacco use and prevent cardiovascular disease. Health Educ Q. 1995;22:478–98.

8. Cenko C, Pulvirenti M. Politics of Evidence: The Communication of Evidence by “Stakeholders’’ when Advocating for Tobacco Point-of-sale Display Bans in Australia. Australian Journal of Public Administration. 2015; doi:10.1111/1467-8500.12138.

9. Chandora R, Song Y, Chaussard M, Palipudi KM, Lee KA, Ramanandraibe N, et al. Youth access to cigarettes in six sub-Saharan African countries. Prev Med. 2016; doi: 10.1016/j.ypmed.2016.01.018.

10. Chriqui JF, Ribisl KM, Wallace RM, Williams RS, O’Connor JC, el Arculli R. A comprehensive review of state laws governing Internet and other delivery sales of cigarettes in the United States. Nicotine Tob Res. 2008; doi:10.1080/14622200701838232.

11. Cummings KM, Pechacek T, Shopland D. The Illegal Sale of Cigarettes to United-States Minors - Estimates by State. Am J Public Health. 1994; doi:10.2105/AJPH.84.2.300.

12. Difranza JR, Savageau JA, Aisquith BF. Youth access to tobacco: The effects of age, gender, vending machine locks, and “it’s the law” programs. Am J Public Health. 1996;86:221–4.

13. Emmons KM, Kawachi I, Barclay G. Tobacco control: a brief review of its history and prospects for the future. Hematol Oncol Clin North Am. 1997; doi:10.1016/S0889-8588(05)70425-1.

14. Fichtenberg CM, Glantz SA. Youth access interventions do not affect youth smoking. Pediatrics. 2002;109:1088–92.

15. Forster JL, Hourigan ME, Kelder S. Locking devices on cigarette vending machines: evaluation of a city ordinance. Am J Public Health. 1992;82:1217–9.

16. Forster JL, Murray DM, Wolfson M, Blaine TM, Wagenaar AC, Hennrikus DJ. The effects of community policies to reduce youth access to tobacco. Am J Public Health. 1998;88:1193–8.

17. Forster JL, Widome R, Bernat DH. Policy interventions and surveillance as strategies to prevent tobacco use in adolescents and young adults. Am J Prev Med. 2007; doi:10.1016/j.amepre.2007.09.014.

18. Forster JL, Wolfson M. Youth access to tobacco: policies and politics. Annu Rev Public Health. 1998; doi:10.1146/annurev.publhealth.19.1.203.

19. Gilpin EA, Lee L, Pierce JP. Does adolescent perception of difficulty in getting cigarettes deter experimentation? Prev Med. 2004; doi:10.1016/j.ypmed.2003.12.001.

20. Gottlieb NH, Goldstein AO, Flynn BS, Cohen EJE, Bauman KE, Solomon LJ, et al. State legislators’ beliefs about legislation that restricts youth access to tobacco products. Health Educ Behav. 2003; doi:10.1177/1090198102251033.

21. Gratias EJ, Krowchuk DP, Lawless MR, Durant RH. Middle school students’ sources of acquiring cigarettes and requests for proof of age. J Adolesc Health. 1999; doi:10.1016/S1054-139X(99)00019-1.

22. Grucza RA, Plunk AD, Hipp PR, Cavazos-Rehg P, Krauss MJ, Brownson RC, et al. Long-term effects of laws governing youth access to tobacco. Am J Public Health. 2013; doi:10.2105/AJPH.2012.301123.

23. Hagquist C, Sundh M, Eriksson C. Smoking habits before and after the introduction of a minimum-age law for tobacco purchase: analysis of data on adolescents from three regions of Sweden. Scand J Public Health. 2007; doi:10.1080/14034940701256925.

24. Jacobson PD, Wasserman J. The implementation and enforcement of tobacco control laws: Policy implications for activists and the industry. J Health Polit Policy Law. 1999; doi:10.1215/03616878-24-3-567.

25. Kanda H, Osaki Y, Ohida T, Kaneita Y, Munezawa T. Age verification cards fail to fully prevent minors from accessing tobacco products. Tob Control. 2011; doi:10.1136/tc.2010.036947.

26. Kirchner TR, Villanti AC, Cantrell J, Anesetti-Rothermel A, Ganz O, Conway KP, et al. Tobacco retail outlet advertising practices and proximity to schools, parks and public housing affect Synar underage sales violations in Washington, DC. Tob Control; doi:10.1136/tobaccocontrol-2013-051239.

27. Landrine H, Klonoff EA. Validity of assessments of youth access to tobacco: the familiarity effect. Am J Public Health. 2003; doi:10.2105/AJPH.93.11.1883.

28. Lee S, Ling PM, Glantz SA. The vector of the tobacco epidemic: tobacco industry practices in low and middle-income countries. Cancer Causes Control. 2012; doi:10.1007/s10552-012-9914-0.

29. Levy DT, Chaloupka F, Slater S. Expert opinions on optimal enforcement of minimum purchase age laws for tobacco. J Public Health Manag Pract. 2000; doi:10.1097/00124784-200006030-00015.

30. Liang L, Chaloupka F, Nichter M, Clayton R. Prices, policies and youth smoking, May 2001. Addiction. 2003;98:105–22.

31. Mamudu HM, Veeranki SP, John RM. Tobacco use among school-going adolescents (11-17 years) in Ghana. Nicotine Tob Res. 2013; doi:10.1093/ntr/nts269.

32. Millett C, Lee JT, Gibbons DC, Glantz SA. Increasing the age for the legal purchase of tobacco in England: impacts on socio-economic disparities in youth smoking. Thorax. 2011; doi:10.1136/thx.2010.154963.

33. Moodie R, Stuckler D, Monteiro C, Sheron N, Neal B, Thamarangsi T, et al. Profits and pandemics: Prevention of harmful effects of tobacco, alcohol, and ultra-processed food and drink industries. Lancet. 2013; doi:10.1016/S0140-6736(12)62089-3.

34. Morain SR, Winickoff JP, Mello MM. Have tobacco 21 laws come of age? N Engl J Med. 2016; doi:10.1056/NEJMp1603294.

35. Mosher JF. The merchants, not the customers: resisting the alcohol and tobacco industries’ strategy to blame young people for illegal alcohol and tobacco sales. J Public Health Policy. 1995;16:412–32.

36. Nagler RH, Viswanath K. Implementation and research priorities for FCTC Articles 13 and 16: tobacco advertising, promotion, and sponsorship and sales to and by minors. Nicotine Tob Res. 2013; doi:10.1093/ntr/nts331.

37. Ribisl KM, Williams RS, Gizlice Z, Herring AH. Effectiveness of state and federal government agreements with major credit card and shipping companies to block illegal Internet cigarette sales. PLoS One. 2011; doi:10.1371/journal.pone.0016754.g002.

38. Rimpelä AH, Rainio SU. The effectiveness of tobacco sales ban to minors: the case of Finland. Tob Control. 2004;13:167–74.

39. Robertson L, Cameron C, McGee R, Marsh L, Hoek J. Point-of-sale tobacco promotion and youth smoking: a meta-analysis. Tob Control. 2016; doi:10.1136/tobaccocontrol-2015-052586.

40. Schneider S, Meyer C, Yamamoto S, Solle D. Implementation of electronic locking devices for adolescents at German tobacco vending machines: intended and unintended changes of supply and demand. Tob Control. 2009; doi:10.1136/tc.2008.028035.

41. Schneider S, Gruber J, Yamamoto S, Weidmann C. What Happens After the Implementation of Electronic Locking Devices for Adolescents at Cigarette Vending Machines? A Natural Longitudinal Experiment From 2005 to 2009 in Germany. Nicotine Tob Res. 2011; doi:10.1093/ntr/ntr067.

42. Sebrie EM, Glantz SA. Attempts to undermine tobacco control: tobacco industry “youth smoking prevention” programs to undermine meaningful tobacco control in Latin America. Am J Public Health. 2007; doi:10.2105/AJPH.2006.094128.

43. Sharma LL, Teret SP, Brownell KD. The food industry and self-regulation: standards to promote success and to avoid public health failures. Am J Public Health. 2010; doi:10.2105/AJPH.2009.

44. Shelton DM, Alciati MH, Chang MM, Fishman JA, Fues LA, Michaels J, et al. State laws on tobacco control--United States, 1995. MMWR CDC Surveill Summ. 1995;44:1–28.

45. Spivak AL, Monnat SM. Prohibiting juvenile access to tobacco: Violation rates, cigarette sales, and youth smoking. Int J Drug Policy. 2015; doi:10.1016/j.drugpo.2015.03.006.

46. Sundh M, Hagquist C. Compliance with a minimum-age law of 18 for the purchase of tobacco--the case of Sweden. Health Educ Res. 2006; doi:10.1093/her/cyl007.

47. Tauras JA. Differential impact of state tobacco control policies among race and ethnic groups. Addiction. 2007; doi:10.1111/j.1360-0443.2007.01960.x.

48. Conley Thomson C, Hamilton WL, Siegel MB, Biener L, Rigotti NA. Effect of local youth-access regulations on progression to established smoking among youths in Massachusetts. Tob Control. 2007; doi:10.1136/tc.2006.018002.

49. Wakefield M, McLeod K, Perry CL. “Stay away from them until you’re old enough to make a decision”: tobacco company testimony about youth smoking initiation. Tob Control. 2006; doi:10.1136/tc.2005.011536.

50. Weinbaum Z, Quinn V, Rogers T, Roeseler A. Store tobacco policies: a survey of store managers, California, 1996-1997. Tob Control. 1999;8:306–10.

51. White MM, Gilpin EA, Emery SL, Pierce JP. Facilitating adolescent smoking: who provides the cigarettes? Am J Health Promot. 2005; doi:10.4278/0890-1171-19.5.355.

52. Whyte G, Gendall P, Hoek J. Advancing the retail endgame: public perceptions of retail policy interventions. Tob Control. 2014; doi:10.1136/tobaccocontrol-2013-051065.

53. Williams RS, Ribisl KM. Internet cigarette vendor compliance with credit card payment and shipping bans. Nicotine Tob Res. 2014; doi:10.1093/ntr/ntt159.
